# Generation and analysis of expressed sequence tags (ESTs) for marker development in yam (*Dioscorea alata *L.)

**DOI:** 10.1186/1471-2164-12-100

**Published:** 2011-02-09

**Authors:** Satya S Narina, Ramesh Buyyarapu, Kameswara Rao Kottapalli, Alieu M Sartie, Mohamed I Ali, Asiedu Robert, Mignouna JD Hodeba, Brian L Sayre, Brian E Scheffler

**Affiliations:** 1 USDA-ARS, Stoneville, MS, USA; 2 Dow AgroSciences, Indianapolis, IN, USA; 3 Texas Tech University, Department of Plant and Soil Science, Lubbock, Texas 79409, USA; 4 International Institute for Tropical Agriculture (IITA), Oyo Road, PMB 5320 Ibadan, Nigeria; 5 USDA-NIFA, Washington D.C., USA; 6 African Agricultural Technology Foundation, Nairobi 00100, Kenya; 7 Virginia State University, Petersburg, VA23806, USA

## Abstract

**Background:**

Anthracnose (*Colletotrichum gloeosporioides*) is a major limiting factor in the production of yam (*Dioscorea *spp.) worldwide. Availability of high quality sequence information is necessary for designing molecular markers associated with resistance. However, very limited sequence information pertaining to yam is available at public genome databases. Therefore, this collaborative project was developed for genetic improvement and germplasm characterization of yams using molecular markers. The current investigation is focused on studying gene expression, by large scale generation of ESTs, from one susceptible (TDa 95-0310) and two resistant yam genotypes (TDa 87-01091, TDa 95-0328) challenged with the fungus. Total RNA was isolated from young leaves of resistant and susceptible genotypes and cDNA libraries were sequenced using Roche 454 technology.

**Results:**

A total of 44,757 EST sequences were generated from the cDNA libraries of the resistant and susceptible genotypes. Greater than 56% of ESTs were annotated using MapMan Mercator tool and Blast2GO search tools. Gene annotations were used to characterize the transcriptome in yam and also perform a differential gene expression analysis between the resistant and susceptible EST datasets. Mining for SSRs in the ESTs revealed 1702 unique sequences containing SSRs and 1705 SSR markers were designed using those sequences.

**Conclusion:**

We have developed a comprehensive annotated transcriptome data set in yam to enrich the EST information in public databases. cDNA libraries were constructed from anthracnose fungus challenged leaf tissues for transcriptome characterization, and differential gene expression analysis. Thus, it helped in identifying unique transcripts in each library for disease resistance. These EST resources provide the basis for future microarray development, marker validation, genetic linkage mapping and QTL analysis in *Dioscorea *species.

## Background

Yams (*Dioscorea *spp.) are the primary agricultural commodities and major staple crop in Africa. Yam tubers are nutritionally rich and a major source of dietary fiber, carbohydrates, vitamin C and essential minerals. Worldwide, yam consumption is 18 million tons http://www.IITA.org. In 2007, yam production was 52 million tons worldwide, of which Africa produced 96%, and Nigeria is the major producer (71%) with more than 37 *million *tons [1]. The consumer demand for yam is very high in sub-Saharan region of Africa, but the yam production is declining in this region due to factors including anthracnose disease caused by a fungus, *Colletotrichum gloeosporioides *[2], pests, and decline in soil fertility [3]. Yams are polyploid crop species and are propagated vegetatively from tubers (whole or setts).

The water yam (*D. alata*) is the most widely cultivated species and is highly susceptible to anthracnose disease [4,5]. The genetic improvement of yam at IITA and CTCRI (India) concentrated on the development of disease resistant and high yielding varieties. Through classical breeding, it would be very difficult to develop a resistant cultivar due to constraints such as the long growth cycle (8-10 months), dioecious and poor flowering nature, polyploidy, vegetative propagation and heterozygous genetic background [6]. A large collection of germplasm with huge genetic variability is available, and it would be profitable to use candidate gene approach and trap the important trait information for disease and pest resistance.

*Colletotrichum *is a large genus of ascomycete fungi, containing many species which cause anthracnose or blight on a wide range of important crops and ornamental plants [7]. Two genes, *clk1 *which encodes a serine/threonine kinase in *C. lindemuthianum *[8], and *cap20 *which encodes a wall glycoprotein of *C. gloeosporioides appressoria *[9], have been shown to have a role in pathogenicity and virulence, respectively. Necrotrophy is clearly linked to the increased expression of plant cell wall degrading enzymes such as endo-polygalacturonases (endo- PG) and pectin lyases. Previous studies [10] showed that endo-PG, two forms of pectin lyase, a- and b-galactopyranosidase, a-arabinofuranosidase, and a protease are secreted into culture medium containing polypectate of bean cell walls. Pectin lyase activity was first observed 4 days after inoculation of beans with *C. lindemuthianum*, rising to maximum activity at 7 days, after which activity declined. Thus, the expression of pectin lyase activity correlates well with the onset of necrotrophy and the subsequent development of lesions [11]. Therefore the yam leaf material for current investigation was collected on 3rd and 7th day after inoculation with *C. gloeosporioides *fungus.

Initial genetic inheritance studies showed that resistance to yam anthracnose in water yam is dominant and quantitatively inherited [12]. A single major dominant locus controlling resistance in the breeding line TDa 95-0328 was tentatively designated Dcg-1, until allelism was investigated [6]. Lower number of molecular markers in yam limits the genetic mapping efforts. Previously developed SSRs from *Dioscorea tokoro *[13], a wild diploid yam species of East Asia and Japan were found not to be useful in cultivated yams *D. rotundata *and *D. alata *[4]. Most of the current molecular markers for the yam genome by Mignouna *et al*. are based on AFLP and RAPD [14,15]. However, for effective gene discovery and marker-assisted breeding, it is important to develop more user-friendly, efficient, transportable and co-dominant markers such as simple sequence repeat (SSR) markers and single nulceotide polymorphism markers (SNPs). EST analysis is the most efficient and effective approach for the identification of candidate genes and also assist in new molecular markers such as EST-SSRs and SNPs [16].

As of 10^th ^October 2010, there were only 31 EST sequences stored at GenBank (National Center for Biotechnology and Information, http://www.ncbi.nlm.nih.gov/) for the genus *Dioscorea*. To understand the transcriptome of yam and for new marker discovery to assist in yam crop improvement programs, generation of ESTs would be extremely desirable. With the objective of characterizing transcriptome in yam, initial efforts were focused on successful isolation of mRNA, cDNA library construction and sequencing using Sanger's method [17]. Two hundred EST sequences were generated using this approach, but reported little functional significance during BLAST search analysis. To enrich and characterize expressed gene sequence information especially during the anthracnose infection for candidate gene identification and also to utilize the sequence information for new marker discovery, a collaborative effort to generate large number of ESTs was initiated between VSU, USA and IITA, Africa. For this purpose, two anthracnose resistant germplasm lines 87-01091, 95-00328 and a susceptible line 95-0310 were selected for cDNA library construction from leaf tissues inoculated with the *C. gloeosporioides *fungus. The Roche 454 pyrosequencing technology was used to generate EST sequence information (Agencourt Biosciences, MA).

## Results and Discussion

### Generation of ESTs

Assembly process generated 15,196 ESTs in TDa 95-0328; 15,984 ESTs in TDa 95-0310, and 13,577 ESTs in TDa 87-01091 with average sequence lengths of 426, 411 and 524 bases, respectively (Table 1). All the EST sequences generated were submitted to GenBank at NCBI and were assigned Genbank Accession numbers HO809681-HO825421 with dbEST id from 71421255 to 71436995 for library TDa 95-0310; HO825422-HO840419 with dbEST ids from 71436996 to 71451993 for library TDa 95-0328; HO850622 to HO864016 with dbEST ids from 71462196 to 71475590 for library TDa 87-01091. These sequences contributed to >99% of ESTs currently available in yam at Genbank and would serve as the major resource for the yam research community.

**Table 1:** List of Yam Genotypes used, average EST length and total number of ESTs generated in the present study.

Genotype	Special characters	Total number of ESTs identified	Average EST length	Total number of repeats found	Number of ESTs containing SSR sequences
TDa 95-0310	Susceptible to FGS and SGG strains of *C. gloeosporioides*	15984	411	1850	572

TDa 95-0328	Resistant to FGS and susceptible to SGG strain	15196	328	1704	556

TDa 87-01091	Susceptible to FGS and Resistant to SGG strain	13577	524	2424	574

### Transcriptome Analysis

**Figure 1:**
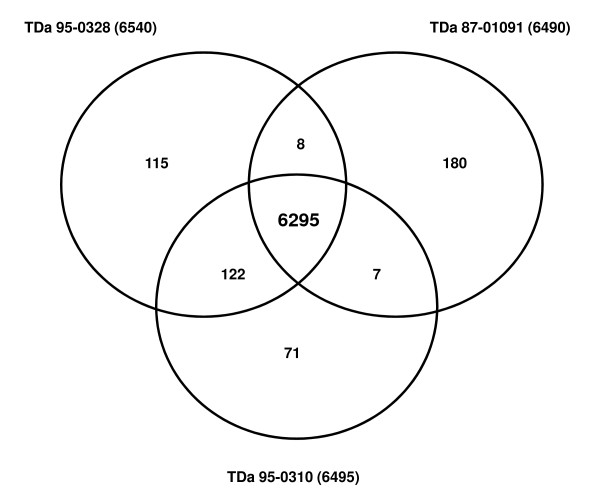
**Venn Diagram describing distribution of ESTs across three libraries, TDa 95-0310, TDa 95-0328, and TDa 87-01091**.

EST sequences obtained from each individual library were first analyzed using Blast2GO http://blast2go.org[18]. Blast2GO provides the gene annotation information for the ESTs and also helps in comparison of two EST datasets to find expression differences. BLAST search analysis across all three libraries revealed highest homology with the sequences from *Oryza sativa*, *Vitis vinifera*, *Populus trichocarpa *and other crop species. Mapping and annotation steps in Blast2GO program resulted in providing GO IDs (Gene Ontology identifiers) to individual ESTs in each dataset of the three libraries (Additional file 1). A total of 9,133 (57.86%) of 15,787 ESTs were annotated with 4,074 non-redundant (nr) GO IDs in the susceptible TDa 95-0310 germplasm line, while in resistant TDa 95-0328 line, 8,985 (59.75%) of 15,038 ESTs were annotated with 3,839 nr GO IDs and in other resistant line TDa 87-01091 library, 4,577 (34.08%) of 13,429 ESTs were annotated with 2,465 nr GO IDs. Approximately 50% of ESTs could not be annotated and remained unknown using Blas2GO. To complement and comprehensively annotate the Yam transcriptome, all the EST sequences were submitted to Mercator tool of MapMan database. Mercator tool generated functional predictions by searching 6 reference databases (3 BLAST based and 2 based on reverse position-specific BLAST and InterProScan) and assigned MapMan Bin Ids (additional file 2). Still 44% of ESTs lacked significant homology with genes in other crops in each of the three datasets and these may include novel metabolic genes in yam. A comparative analysis was done using the Bin ID information from each library to identify unique ESTs in each library and also the common genes shared with other libraries. The distribution of ESTs with known function is presented as a Venn diagram in Figure 1. A large subset of 6295 ESTs representing 660 unique Bin IDs were shared across three libraries and these may account for housekeeping genes involved in general cellular metabolism. The two resistant lines TDa 95-0328 and TDa 87-01091 had 115 and 180 unique ESTs, respectively, which may account for the tolerance against *C. gloeosporioides *fungus.

**Figure 2:**
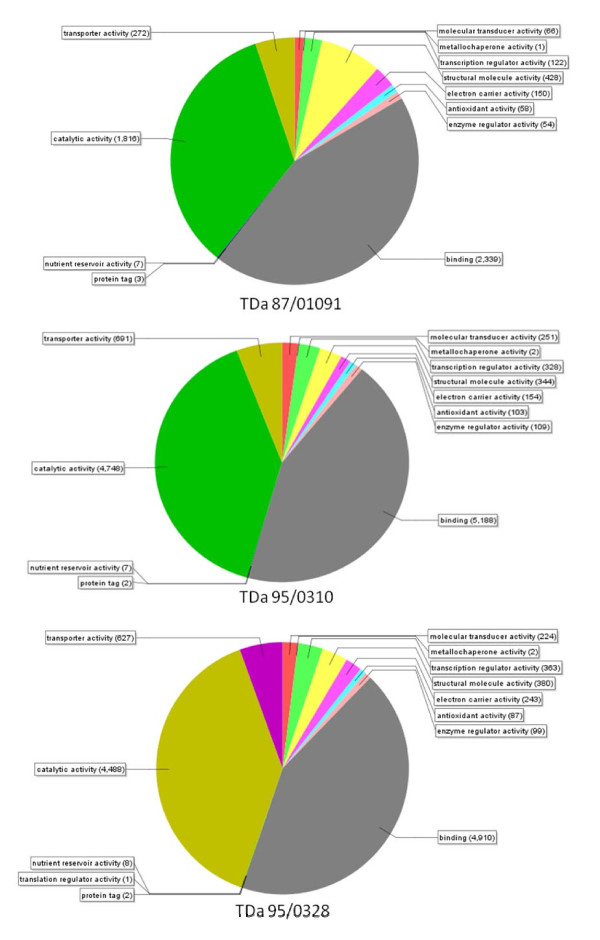
**Distribution of unique ESTs into MapMan functional categories in the three yam genotypes**.

In TDa 95-0310 susceptible genotype, there were 71 unique sequences which predominantly matched genes for protein synthesis and nucleotide synthesis (Figure 2). However, there were several sequences annotated as protein kinases and were known to have a definite role in disease signaling [19]. Another interesting feature is the presence of genes for synthesis of cell wall precursors and secondary metabolites which might be highly induced due to severe biotic stress. There were also genes for recycling NAD to maintain glycolysis and substrate level phosphorylation in the absence of oxygen in damaged leaf of susceptible genotype [20]. Removal of the toxic compounds like proline is very essential for plant survival under stress [21]. There were unique genes for proline degradation in susceptible genotype suggesting the presence of a possible mechanism of detoxification during pathogenesis.

Common sequences between TDa 95-0310 and TDa 95-0328, were annotated as 122 genes representing cell wall precursors, co-factor and vitamin metabolism, tetrapyrrole synthesis, C1-metabolism, lipid metabolism, amino acid metabolism, secondary metabolism, hormone metabolism, major and minor CHO metabolism, nucleotide metabolism, genes for regulation of transcription, amino acid activation, protein synthesis, protein degradation, energy metabolism, and genes for sugar and light signaling. On the other hand, the common genes between TDa 95-0328 and TDa 87-01091 were cell wall proteins, sulfate assimilation related genes, phosphatidylserine decarboxylase, hydroxymethylpyrimidine kinase, L-Galactono-1,4-lactone dehydrogenase, genes for dicer degradation, GeBP like transcription factor, and myo inositol oxygenase. Similarly, TDa 87-01091 and TDa 95-0310 shared sequences related to dihydroneopterin aldolase, Glycine cleavage H protein, phosphomannose isomerase, C-lectin, alcohol dehydrogenase, and malate dehydrogenase genes. Although these genes have specific role in metabolism, regulation and signaling, the significance of their sharing between yam genotypes cannot be speculated and is beyond the scope of this paper.

### Functional Analysis

**Figure 3:**
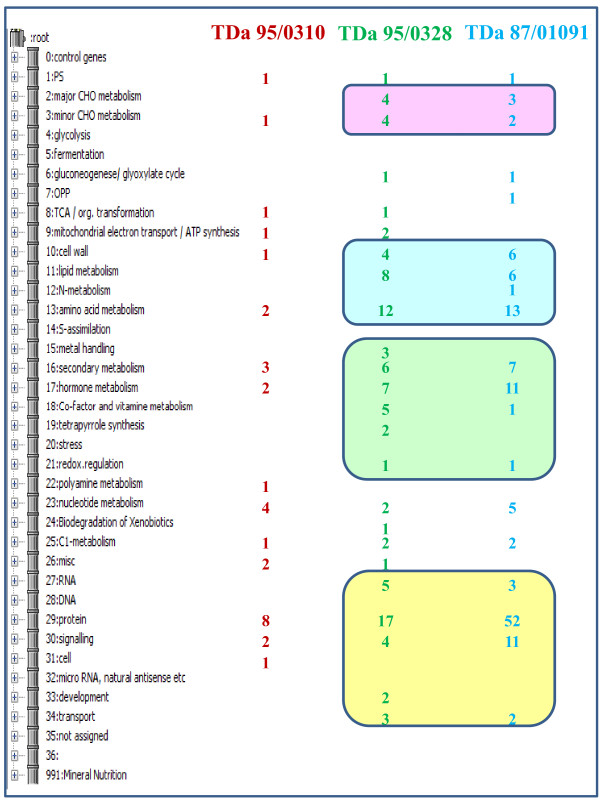
**GO distribution in TDa 95-0310, TDa 95-0328 and TDa 87-01091 based on molecular function**.

Using the GO ID information, the ESTs were classified into subsets based on molecular function and is represented as a pie diagram for each library (Figure 3). A major subset of ESTs (> 60%) across all libraries was linked to binding and catalytic activity, where the remaining groups involved transporter, signal transduction, transcription factors, secondary metabolism and antioxidant activities. Based on biological process classification, a major proportion of the genes were involved in oxidation-reduction activity across all libraries and these may involve genes in energy metabolisms and other housekeeping activities. ESTs were also grouped under other biological processes such as photosynthesis, membrane transport, transcription, translation, protein folding, and others. However, a significant proportion of the expressed genes (~5-10%) were biotic stress and other stress responsive genes (Figure 3).

### Differential gene expression analysis

From the EST datasets, it is interesting to identify genes involved in defense mechanisms that were differentially expressed in susceptible and resistant yam genotypes. Using the Bin Ids of ESTs in each library, the expression of various ESTs were compared from one library to the other. These results were summarized in Figure 2 and additional file 3. The resistant genotypes had unique ESTs related to carbohydrate metabolism, cell wall biogenesis, lipid and amino acid metabolism, secondary and hormone metabolism, transcription factors, protein synthesis, and signaling proteins (Figure 2). Even though, the EST datasets is not comprehensive of the entire yam transcriptome, this analysis would shed some light on the gene expression differences during anthracnose infection. Further investigation of these differentially expressed genes using microarray platforms or RNA-seq or real-time quantitative PCR experiments, would assist in elucidating the underlying molecular mechanism for anthracnose tolerance in yam.

### Informative defense related genes during anthracnose infection

The differential gene expression analysis provided the gene information that might be potentially over-expressed or under-expressed compared to other libraries. These results were summated in additional file 3.

Multiple pathogenesis-related genes and host defense related genes including signaling genes were identified from the ESTs in the resistant lines and they include *nicotiana* lesion inducing proteins, *erwinia* induced proteins, cysteine proteases, cysteine protease inhibitors, peroxidases, extensin, wall associated kinases, brassinosteroid receptor kinases, ethylene responsive genes, phosphatidylinositol 4-kinase, leucine rich repeat (LRR) genes, serine-threonine protein kinases and others signaling proteins. Signal transduction in plants, leading to the expression of defense genes, is initiated by leucine rich repeat (LRR)-type membrane receptors containing intrinsic kinase activity as in the case of FLS2 [22].

There were unique genes involved in isoprenoid and flavonoids biosynthesis and fatty acid synthesis and elongase activity in TDa 87-01091 compared to TDa 95-0310 and TDa 95-0328. There were hits specifically showing homologies to peroxisome biogenesis protein pex1 (GO: 0009851, GO: 0017111) and perxisome assembly factor (GO: 0016558) in library 87 and 328. This confirms previous reports of plant peroxisomes role in the biosynthesis of the signaling molecules like jasmonic acid, β-oxidation of indole butyric acid (IBA), and sulphur and polyamine metabolism. Moreover, evidence is emerging from recent studies that peroxisomes have important functions in specific defense mechanisms, conferring resistance against pathogen attack [23].

There were homologies to 12-oxo-phytodienoic acid reductases (OPRs), enzymes of the octadecanoid pathway, which convert linolenic acid to a phytohormone jasmonic acid. Fifty nine significant matches to auxin responsive proteins (GO: 0006417, 9725, 9734, 46983), auxin signaling f-box 3 (GO: 0002237) and auxin response transcription factors (GO: 0045449) were observed in TDa 87-01091 and TDa 95-0328, confirming their role in host defense against pathogens.

Actually Plant peroxidases (POXs) transduce the extracellular signals into the redox signals that eventually stimulate the intracellular Ca2+ signaling required for induction of defense responses [24]. There were significant hits to different plant peroxidases in resistant libraries confirming their requirement for defense against *Colletotrichum *fungus.

The hits to pectate lyase were few, one each in TDa 95-0328 (GO: 30570) and TDa 87-01091 (GO: 0016829) compared to four in TDa 95/0310. The contribution of a single Pectate lyase gene (*pel*) to the pathogenic abilities of *C. magna *confirmed that Pectate lyase is a pathogenicity factor required for the penetration and colonization of *Colletotrichum *species [25]. Cell-wall-degrading enzymes (CWDEs), like pectolytic enzymes, are considered to play a role in the pathogenesis of bacteria and fungi on their hosts [26]. The peptidoglycan-binding LysM domain-containing protein and beta-1,4-glucanases may help in fungal cell wall degradation [27] as there were significant hits observed in the resistant genotypes. The disruption of a single enzyme may be complemented by the activity of other CWDEs [28]. There was also homology to chitin binding, chitinase activity and chitin catabolic/metabolic process and were abundant in 87-01091, a genotype resistant to *C. gloeosporioides *strain SGG.

The activation of the defense response, after pathogen inoculation, is the most important factor for the success of host resistance [29]. The EST data from our study also suggest strength of the expression of ubiquitous early acting genes for establishment of the resistance response of the resistant genotype 87-01091.

### Marker discovery

The EST sequence information generated in this study also serves as a major resource to generate new molecular markers. Simple sequence repeat motifs detected in EST sequences are usually termed as EST-SSRs. These are very informative markers as they exist within the gene sequence. A total of 44,254 ESTs from three genotypes, with an average length of 500 bp were used to evaluate for the presence of SSR motifs. There are a total of 1702 EST sequences containing SSRs identified from three libraries. In this work, SSRs were considered for primer design that fitted the following criteria: a minimum pattern length of 12 bp, excluding polyA and polyT repeat, at least 7 repeat units in case of di-nucleotide and at least 5 repeat units for tri-, tetra-, penta- and hexa-nucleotide SSRs. There are about a total of 3459 good quality repeats containing di (289), tri (1271), tetra (66), penta (19), hexa (53) and hepta (3) repeats which were used for designing primers (Table 1). The default settings for Primer3 program: optimum temperature of 63^o^C and an optimum primer size of 24 bases were selected for designing the primers. A total of 1705 primer pairs were designed from 1702 EST sequences. A subset were tested for screening genomic DNA of 3 genotypes using simple 1.2% agarose gel electrophoresis for polymorphism detection and found polymorphic. These SSR primers are summarized in additional file 4.

Identification of homologous SNPs is very challenging in a polyploid species such as yam. HaploSNPer generates putative SNP haplotype for each contig using CAP3 generated .ace file. Therefore, we can verify whether a SNP is heterozygous within each genotype or homozygous at specific loci. If they are heterozygous within each genotype, basically they may be copies from homeologous genomes or paralogous copies. Even though multiple sequence clusters were identified with single nucleotide variations, most of them were polymorphic within a germplasm line at many loci. Usually they will be heterozygous always and exhibit no polymorphism between the genotypes. Selection of such SNPs usually results in false positive assays. However, we were able to detect 104 candidate SNPs between libraries 310 and 328 that are homologous within each genotype. These SNPs can be used for further genotyping and generating genetic maps in yam.

## Conclusion

Despite the economic importance of yam, very little genomic or transcriptome sequence data is publicly available. This is the first large scale EST generation attempt made in yam with the objective of providing a comprehensive annotated transcriptome dataset that will be publicly available, and also used as a resource for novel gene and new marker discovery for crop improvement in yam. We have successfully sequenced transcriptomes of two resistant and one susceptible lines of yam under the conditions of anthracnose infection. Approximately 56% of total ESTs were annotated and analyzed for functional characterization and differential expression of genes for tolerance to anthracnose disease. We also used this dataset as a resource to design new SSR and SNP markers. They serve as a useful tool in identifying genetic variation in the current cultivars, wild relatives and also assist in generating genetic linkage maps. The SSR markers generated are currently being evaluated at IITA for use in their yam improvement program. The identified SNP markers will be validated to identify QTL regions through the ongoing research on yam at IITA, USDA-ARS and VSU. The markers generated could be used on F1 population generated at IITA to develop first generation EST-SSR based QTL map for yam.

## Methods

The *D. alata *genotypes were selected based on their scoring (0-6 scale) to leaf damage by [30] anthracnose for resistance (0-17.5% mean leaf area damage). The fully expanded, pathogen challenged, young leaves from yam genotypes with differential resistance/susceptibility to FGS (fast-growing salmon) and SGG (slow-growing grey) strains of anthracnose (Table 1) were harvested at IITA. The pathogen inoculations were done at IITA following the standardized inoculation procedures [31] similar to the method described by Green *et al*. [32]. A spore suspension of *C. gleosporoides *in sterile distilled water was prepared from 7-10 days old single-spore cultures (based on virulence on *D. alata*) and standardised to 10^6 ^spores per ml with tween 80 (Merck)(1.2% v/v) added as wetting agent. Young leaves of both the genotypes were inoculated on both sides and the green leaf tissues next to the infestation was collected on 4th day and 7th day after infestation.

These leaf samples were freeze dried and supplied to VSU for genomic studies in 2007. Extraction of high quality RNA is very challenging from yam tissues due to high polyphenolic content and other residues. Total RNA was isolated from these leaf samples using the optimized RNA isolation protocol [17] and was quantified using Spectrophotometer (Bio-Rad Laboratoires, CA, USA). The quality was further confirmed by running a 1.2% Formaldehyde Agarose Gel Electrophoresis. The cDNA library was constructed using Clontech's Creator SMART cDNA library construction kit with the pDNR-lib vector (Clonetech Laboratories, CA, USA). The total RNA was reverse transcribed to cDNA using Powerscript Reverse Transcriptase, using kit primers SMART IV Oligonucleotide and CDS III/3' PCR primer. The cDNA was PCR-amplified using the Advantage 2 PCR kit, using the SMART 5' PCR III primer and CDS III/3' PCR primer, using between 18 and 26 cycles according to the manufacturer's instructions. The libraries from all the genotypes were arrayed in 96 well plates and stored at -80°C.

To assess the quality of the library, electro competent cells, DH10B *E. coli *bacterial cells (T1 phage resistant) were transformed with the vector containing cDNA inserts (Gene pulser, Bio-Rad Laboratoires, CA, USA) grown in liquid suspension for two hours and then plated onto LB agar plates with 30 ug/ml chloramphenicol. The independent colonies were collected separately and arrayed into 96 well plates for Sanger sequencing. Preliminary sequencing efforts revealed very little information about disease resistance due to less number of hits to functional genes; we focused our efforts to next generation sequencing methods. The cDNA library was constructed for two resistant and one susceptible line. The cDNA quality was tested by random cloning of the sequences using creator SMART cDNA library construction kit (Clonetech Laboratories, CA) and individual clones were sequenced using Sanger sequencing method. Agencourt Bioscience Corporation (MA, USA) was identified as a preferred partner to sequence the cDNA libraries from pathogen challenged leaf tissues using Roche 454 pyrosequencing technologies. High quality fragment cDNA library was constructed and 454 sequence data was generated by Agencourt Biosciences (MA, USA). Raw sequence data generated from three cDNA libraries using 454 GS FLX (Titanium chemistry) instrument was assembled into contigs to generate EST dataset for each library (Agencourt Biosciences, MA). Sequencing of these libraries from the challenged leaves of resistant and susceptible genotypes generated a total of 195,937 (TDa 87-01091), 217,868(TDa 95-00328) and 399,987(TDa 95-0310) cDNA raw reads, respectively.

### Sequence assembly, analysis and annotation

a. Preliminary sequencing data analysis: The Sanger sequences were analyzed using NCBI BLAST tool. The vector sequences from each sequence were trimmed manually, Sequences less than 150 base pairs were removed and then ESTs were identified based on NCBI "blastn" similarities, Blast hits considered significant cover at least 150 bases with 80-96% identity. All ESTs sharing significant similarities were clustered together. ESTs with no significant similarities to any other ESTs were given their own ID number and referred as singletons.

b. 454 sequence data analysis: The sequencing agency provided high quality raw reads as well as aligned EST sequences for three libraries. These sequences were analyzed using Blast2GO Program. BLAST hits with an e-value of 10^-5 ^or less, which corresponds approximately to a 60-bp contiguous perfect match in the data set, were considered to be successful hits against the transcriptome. The aligned EST Sequences with BlastX hits were mapped and annotated according to gene ontology terms (GO) using the program Blast2Go [33]. The distribution of genes in each ontology categories was examined and the percentages of unique sequences in each of the assigned GO term namely, biological process, molecular function, and cellular component, were computed and presented. Using the available annotated EST information in each library, a Fisher exact test was performed at p-value 0.05 in Blast2GO program to compare the expression level of various ESTs from one library to the other [18].

c. Mapman analysis: For data analysis by MapMan software version 3.5.0 BETA [34]http://gabi.rzpd.de/projects/MapMan/, the EST sequences from yam libraries were uploaded into Mercator tools and the mapping file was generated. Functional predictions and Bin Ids were generated by searching a variety of reference databases (currently 6 are available: 3 BLAST-based, 2 reverse position-specific BLAST based and InterProScan) and subsequently evaluating and compiling the search results for each input gene to propose a functional Bin based on the manually curated binning of the reference database entries. List of ESTs represented in the mapping file and their corresponding Bin identifiers are listed in additional file 2.

d. BLAST search against *Colletotrichum *genes: The total yam EST sequences were blasted against 962 nucleotide sequences available at NCBI for *C. gloeosporioides *(as of October 17, 2010). There were not any significant hits from the yam ESTs (e-value <10^-15^) except a few that belong to 18 s ribosomal unit of *Colletotrichum *and the rest being the calmodulin, actin and EF1-alpha genes. The alignment length of these hits is also less than 50 bp.

e. BLAST search against Plant Vs fungal gene data base: We find very few significant similarities with the fungal genes 1,20,8, respectively in TDa 95-0310, TDa 95-0328 and TDa 87-01091 with their alignment length ranging from 637-917 at eValue = 0 and BIT score more than 100. There were 12,296 genes unique to yam not showing hits to either plant or fungal genes while 31,561 ESTs showing similarities to plant genes. These 29 ESTS were eliminated for final EST analysis using Mapman.

### Bioinformatics mining of microsatellites

The total ESTs more than 150 base pairs were searched for microsatellites using Schroeder's SSR finder software [35]. In this work, SSRs were considered for primer design that fitted the following criteria: a minimum pattern length of 12 bp, excluding polyA and polyT repeat, at least 7 repeat units in case of di-nucleotide and at least 5 repeat units for tri-, tetra-, penta- and hexa-nucleotide SSRs. The default settings for Primer3 input were optimum temperature of 63^o^C and an optimum primer size of 24 bases.

### Identification of SNPs

EST sequences were pooled based on the GO ID information captured during annotation process using a relational database. These homo or orthologous sequences were submitted to the web-based HaploSNPar program to generate haplotype clusters and identify candidate SNPs with default parameters [35].

## List of abbreviations

RNA: Ribonucleic Acid; DNA: Deoxy Ribonucleic Acid; cDNA: Complementary DNA; BLAST: Basic Local Alignment Search Tool; bp: base pairs; EST: expressed sequence tag; GO: gene ontology; SSR: Simple Sequence Repeat; SNP: Single Nucleotide Polymorphism;

## Authors' contributions

SN conducted the major part of this study, analyzed the data and prepared the manuscript. RB participated in data analysis, manuscript preparation and revisions. KRK analyzed the data using MapMan software, manuscript preparation and reviewed the manuscript. MA supervised the study and reviewed the manuscript. AR and AS supplied the challenged leaf material and participated in manuscript revisions. MH, BLS and BS reviewed the manuscript. All the authors read and approved the final manuscript.

## Supplementary Material

Additional file 1 **Gene Ontology (GO IDs) identifiers for three libraries**. Mapping and annotation steps in Blast2GO program resulted in providing GO IDs to individual ESTs in each dataset of the three libraries, TDa 95-0310, TDa 95-0328 and TDa 87-01091. Click here for file

Additional file 2 **Yam sequences annotated using Mercator tool of MapMan database**. Yam ESTs for three libraries with MapMan annotations were presented in sheet 1, Mapping file generated from Mercator tool with Bin Codes for each yam EST matching to specific genes on sheet 2 and unique Bin IDs identified in each library related to anthracnose were listed on sheet 3 of the file. Click here for file

Additional file 3 **Differential gene expression among the three libraries**. The summary of differential gene expression analysis providing the gene information that might be potentially over-expressed or under-expressed compared to other libraries. The sheet 1 of the file has expression differences between TDa 87-01091 compared with TDa 95-0310 and the sheet 2 has expression differences between TDa 95/0328 compared with TDa 95/0310 while sheet 3 has expression differences between TDa 87-01091 compared with TDa 95-0328. Click here for file

Additional file 4 **Simple Sequence Repeat (SSR) primers generated from the project**. The SSR primers generated from library TDa 95-310, TDa 95-0328 and TDa 87-01091 were respectively presented on sheet 1, 2 and 3 of the file. Click here for file
